# Supplementary material for: Pterostilbene and Probiotic Complex in Chemoprevention of Putative Precursor Lesions for Colorectal Cancer in an Experimental Model of Intestinal Carcinogenesis with 1,2-Dimethylhydrazine
Source: Cancers (Basel). 2023 Apr 21;15(8):2401. doi: 10.3390/cancers15082401 (PMC10136993; doi:10.3390/cancers15082401)

FEDERAL UNIVERSITY OF CEARA
FACULTY OF PHARMACY,
DENTISTRY AND NURSING
DEPARTMENT OF
PHARMACY
PHARMACEUTICS LABORATORY

Pterostilbene (PS) analysis

Chromatographic conditions:

Column: 250 x 4.66 mm (length and internal diameter), packed with silica chemically bonded tooctadecylsilane (C18) groups, with a particle size of 5 µm, kept at room temperature.

Mobile phase: Mixture of acetonitrile (ACN) and water in a ratio of 50:50, in isocratic mode. Flow: 1 mL/min.Detection: UV 310 nm, monitoring the entire UV spectrum using a PDA. Injected sample volume: 20 µL.

Sample treatment

Capsule containing PS, described by the manufacturer (Mental Refresment Lot No. 081216) as having 150 mg in each capsule, was emptied and its content solubilized with acetonitrile using a test tube shaker and sonicator, and the final volume was completed to 50 mL, resulting in a concentration of 3mg/mL. This solution was diluted in acetonitrile to obtain solutions in concentrations of 0.03; 0.15 and 0.3 mg/ml (30, 150 and 300 µg/ml).

Liver

Liver samples removed from animals treated under different conditions during the ongoing experiment (group eating only standard chow and group receiving diet added with PS) were fragmented into small sizes using a scalpel blade and then subjected to sonication extraction. with acetonitrile. The resulting suspension was centrifuged at 8000 rpm for 10 minutes and the supernatant transferred to an eppendorf tube.

Test and results

The samples prepared as described above were manually injected into a high-performance liquid chromatograph (HPLC), initially only the solutions containing the PS so that the chromatogram obtained could be evaluated and, if necessary, adjustments made. As the ACN:water (45:55) mobile phase composition was initially used, the PS retention time was close to 20 minutes and the time for each run was long, but the responses in terms of repeatability and linearity indicated that the method employee was able to detect and even quantify the presence of PS in the injected samples, as shown in Figure S1.

Figure S1. chromatograms of PS solutions at concentrations of 30, 150 and 300 µg /mL.

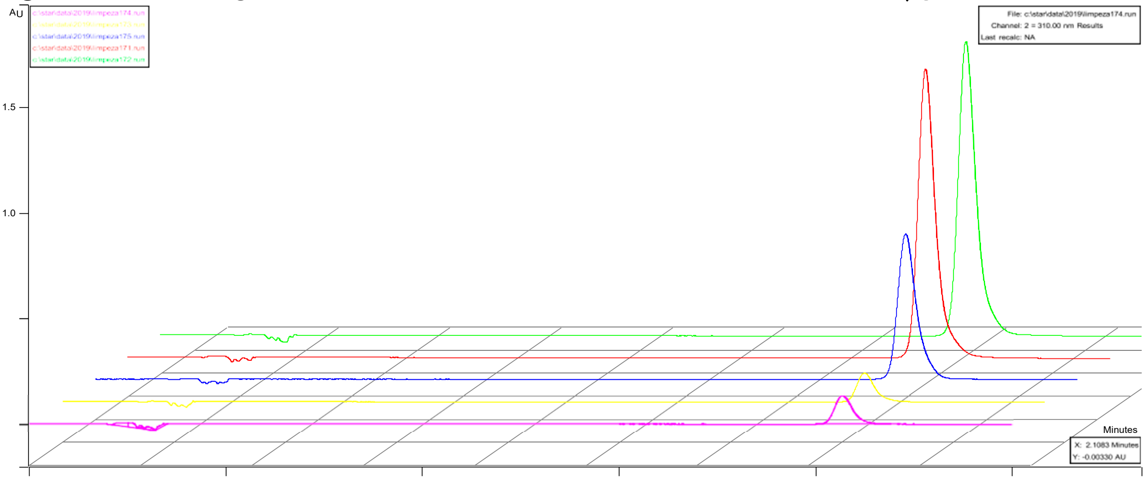

As a result of this result, the composition of the mobile phase with equal parts of ACN and water was used, which reduced the analyte retention time and the total analysis time for each injection.

In Figure S2 we present the results obtained with repeated injections of samples containing 30 µg /mL of the drug in runs with this new composition of the mobile phase. It is observed that the retention time became 11 minutes, and the running time was 15 minutes.

Figure S2. PS chromatograms at 30 µg /mL in a mobile phase consisting of ACN:water (50:50)

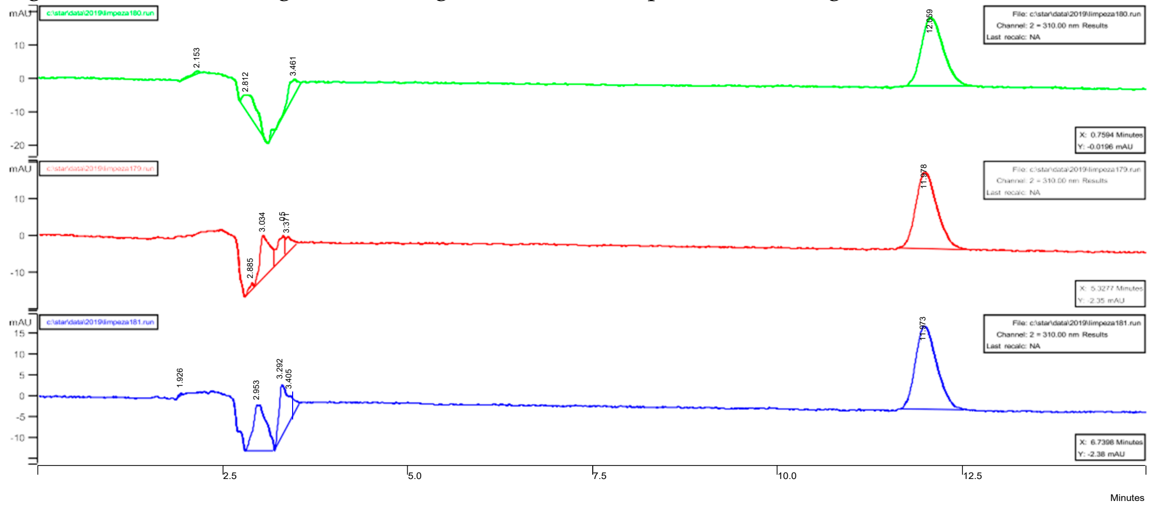

In view of this result, we began to perform injections with samples from the processing of livers, as described above, having processed livers from animals that fed only with feed (standard diet) and animals that received feed containing PS.

Figure S3 shows chromatograms that express the results obtained. The four upper chromatograms show the peak corresponding to the PS at the retention time close to 11 minutes, characterizing the presence of the substance in the sample obtained from the liver extraction of the animals that were on a diet with feed containing PS. Thus, confirming that the ingestion of the drug added to the feed is resulting in its presence in the liver of these animals. The three lower chromatograms represent the samples from the extraction of the livers of the animals submitted to a diet with the normal ration, in which the absence of PS is verified.

Figure S3. chromatograms obtained with ACN extracts from the livers of animals on a normal diet (3 lower) and diet with ration added with PS (4 higher).

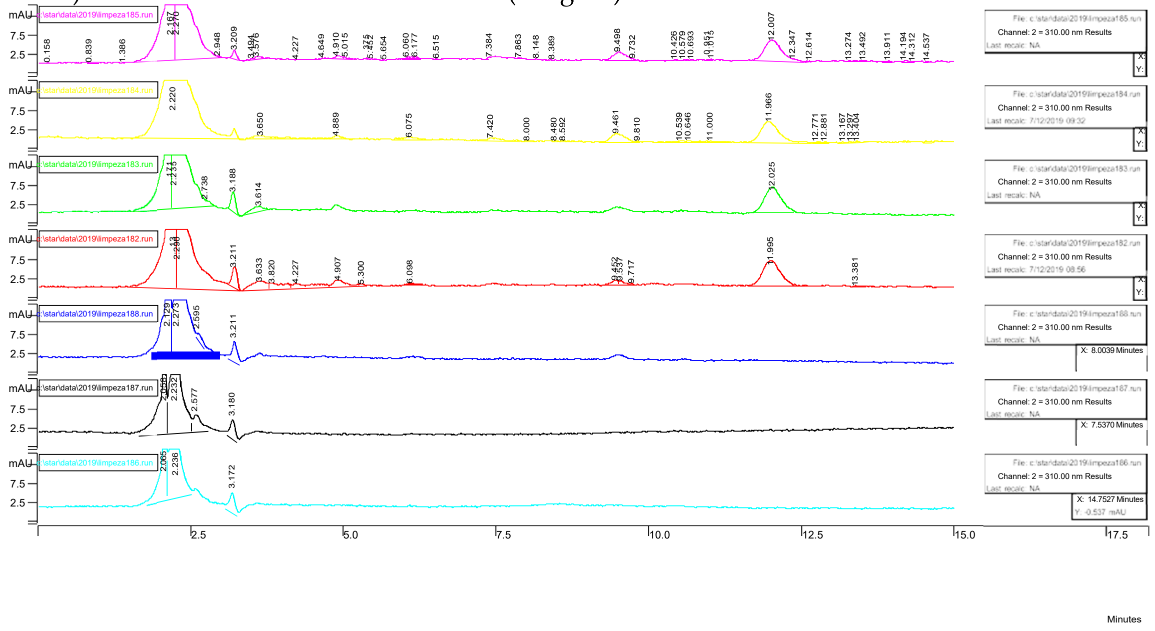

To produce 4000 grams of feed with 300 p.p.m of PS, the process described below was followed. Initially, 4000 grams of feed and 08 PS capsules were separated. The capsules were individually weighed, full and empty, on a high-precision analytical balance (Precise 205 A SCS) (Figure S4A.) and the data obtained used to calculate the average weight of the capsules' contents and the respective standard deviation. The feed used in animal nutrition was reduced to coarse fragments in the cereal mill and then ground to a fine powder using a cyclone rotor mill (TE-651\2 TECNAL) (Figure S4B.) at 1450 rpm using a circular mesh of 0.5 mm in diameter, obtaining the fine powder of the feed as shown in Figure S4C. A pulverized feed concentrate containing PS was prepared by mixing 26 g of the powder from the capsules containing PS with974 g of the powder from the feed. For every 4000 g of feed to be produced, 200 g of this concentrated mixture were used. An aqueous liquid mixture with binding function was prepared by dispersing 20 g of sodium carmellose in distilled water in sufficient quantity to obtain 1 liter, the appearance of which is shown in Figure S4D. To prepare the feed without active ingredient, 4000 g of powdered feed were weighed and a sufficient amount of 2% carmellose sodium binding solution was added to it (Figure S4E). When the appropriate consistency for extrusion was reached, the mass was passed through the extrusion equipment (Figure S4F,G), the pellets obtained being cut into pieces, distributed on trays (Figure S4H) and subjected to drying in an oven with circulation of air at 50°C (Figure S4I).To prepare the ration containing PS, 3800 g of the pulverized ration were mixed with 200 g of the previously prepared concentrate and to the mixture was added, under the action of the planetary mixer, a sufficient amount of the binder solution of carmellose sodium at 2%. When the appropriate consistency for extrusion was achieved, the mass was passed through the equipment, the pellets obtained being cut into pieces, distributed on trays, and dried in an oven with air circulation at 50ºC.

Figure S4. Steps in the production of feed with PS

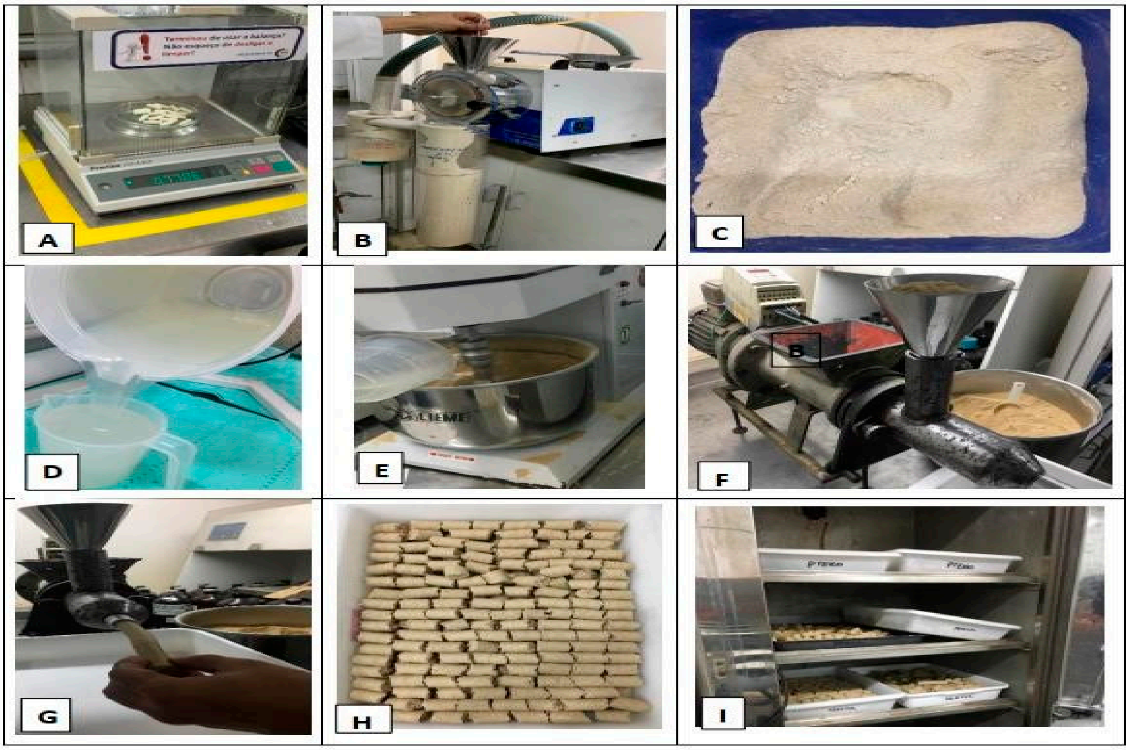

Supplement: Supplementary file 1 [file cancers-15-02401-s001.zip › cancers-2244846-supplementary.pdf]
